# Heterogeneity among Orf Virus Isolates from Goats in Fujian Province, Southern China

**DOI:** 10.1371/journal.pone.0066958

**Published:** 2013-10-15

**Authors:** Xuelin Chi, Xiancheng Zeng, Wenbo Hao, Ming Li, Wei Li, Xiaohong Huang, Shihua Wang, Shuhong Luo

**Affiliations:** 1 College of Life Sciences,Fujian Agriculture and Forestry University, Fuzhou, People's Republic of China; 2 University Key Laboratory for Integrated Chinese Traditional and Western Veterinary Medicine and Animal Healthc re in Fujian Province, College of Animal Sciences, Fujian Agriculture and Forestry University, Fuzhou, People's Republic of China; 3 School of Biotechnology, Southern Medical University, Guangzhou, People's Republic of China; Centers for Disease Control and Prevention, United States of America

## Abstract

Orf virus is a parapoxvirus that causes recurring contagious ecthyma or orf disease in goat, sheep and other wild and domestic ruminants. Infected animals show signs of pustular lesions on the mouth and muzzle and develop scabs over the lesions. Although the infection is usually cleared within 1–2 months, delayed growth and associated secondary infections could still impact the herds. Orf virus can also infect humans, causing lesions similar to the animals in pathological histology. Prior infection of orf virus apparently offers little protective immunity against future infections. Several gene products of orf virus have been identified as responsible for immunomodulatory functions. In our recent study of orf virus isolates from an area along the Minjiang River in northern Fujian Province, we found a high heterogeneity among isolates from 10 farms within a 120-kilometer distance. Only two isolates from locations within 1 km to each other had same viral genes. There is no correlation between the geographical distance between the corresponding collection sites and the phylogenetic distance in ORFV011 or ORV059 genes for any two isolates. This finding suggests that there are diverse populations of orf virus present in the environment. This may in part contribute to the phenomenon of recurring outbreaks and heighten the need for better surveillance.

## Introduction

Orf virus (ORFV), the prototypical species of parapoxviruses (PPV), is the causative agent in the etiology of contagious ecthyma (CE) in sheep and goats. Also known as sore mouth disease and scabby mouth disease, CE is characterized by highly infectious and proliferative lesions, mainly on the lips, tongue, and around the nostrils, but the infection can spread to other non-wooly areas, including the legs, feet and udders [1], [2].Lesions develop with the appearance of vesicles, pustules, papules or nodules that begin to exude clear fluid, but they quickly dry to form crusty scabs, which eventually fall off as the skin underneath heals. CE is a self-limiting infection, usually lasting only 1–2 months [3]. Mortality associated with CE is low, but unless proper care is given to infected animals, mortality can increase, as complications such as fly infestation of affected tissues and secondary infections can be quite common [4]. Depending upon the location of the lesions, infected animals may be unwilling to nurse, eat, or walk [5], and in lactating ewes, udder lesions may also cause mastitis [6]. Infected lambs or kids may need to be hand-fed, as they can transmit the disease by suckling other females. Thus considerable economic losses can occur due to stunted growth or slaughter of the affected animals. CE is also a zoonotic disease that can easily be spread to humans; hands are the most common site of orf infection in humans [7], [8].

Orf virus infections have a worldwide distribution and are ubiquitous in sheep- and goat-raising countries. Outbreaks of orf had been reported by many countries and districts [9]–[13]. This disease has a considerable economic impact on the agricultural sector [14], and it is regarded as one of the most important disease factors affecting the welfare of farmed sheep and goats in developed countries [15]. However, many orf outbreaks go unreported due to the low mortality rate of orf infection in humans. There are very few examples in the literature of epidemiological investigations of orf infections worldwide, and, to date, relatively little data is available concerning orf infection in China. Also, the molecular characterizations of orf isolates have not been fully illustrated. In this study, a multi-faceted investigation was performed to clarify various aspects of epizootic orf infections in the Fujian Province of China. In a serological survey, 349 goat sera from 15 farms were collected to detect humoral antibodies against the orf virus. Based on the distance of average sera titers from different farms, a serum titer clustering tree was constructed to define periods of infection. At the same time, specificities for viral antigens were identified using the Western blotting technique. Also, since vaccination is not used in Fujian Province, a genetic comparison of distinct isolates from this district was conducted to elucidate their molecular characteristics and to facilitate ORFV vaccine development.

## Materials and Methods

### Viral protein purification

The orf virus strain used was NA1/11, which was isolated from a sheep outbreak. The virus was grown in primary Ovine Fetal turbinate (OFTu) cell culture monolayers as described by Wei Li [16]. Cells were harvested when approximately 80–90% of the cells showed cytopathogenic effects (CPE). Cell debris was removed by centrifugation at 1000×g for 10 minutes, and orf virus in the supernatant was purified by sucrose gradient ultracentrifugation [16]. The purified viral particles were heat-inactivated at 96°C for 90 minutes before being disrupted by sonication. The purified native orf viral lysate was then frozen at −80°C until needed for subsequent ELISA and Western blot analyses.

### Sera Collection

**Figure 1:**
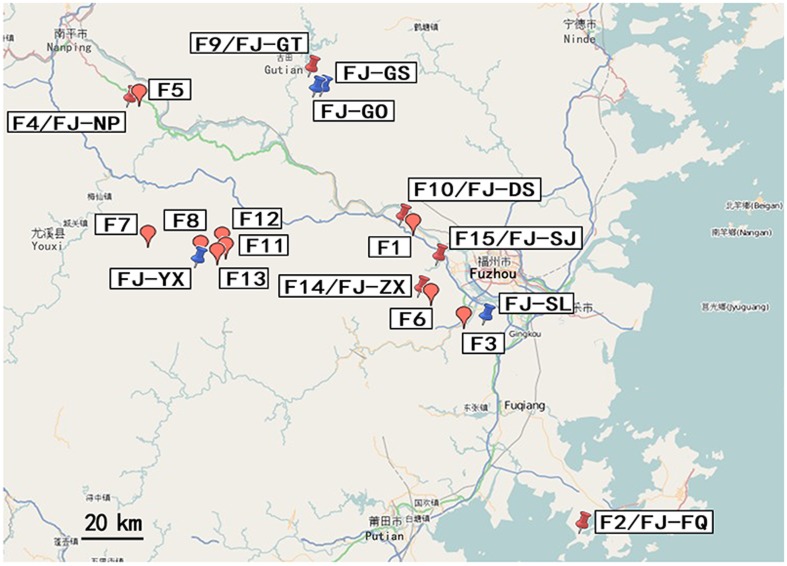
Sites at which samples were collected for investigations of contagious ecthyma and analysis of heterogeneity among orf virus isolates from goats in Northern Fujian Province, China. Red balloon: Sera collection for orf antibodies detection including Farms F1-15. Red thumbtack: Sera collection for orf antibody detection and scab collection for virus isolation and PCR amplification. Blue thumbtack: only scab collection for virus isolation and PCR amplification (http://www.openstreetmap.org/).

All sera were obtained from the Animal Science College, Fujian Agriculture and Forest University, Fujian Province, China, and stored at −80°C. Non-immune sera were obtained from goat flocks that had never been infected with ORFV and were never inoculated with orf vaccine. Test sera were collected from 15 different goat farms in four districts of the Fujian Province of China (Fig.1; Table 1). Among of these farms, four of them were experiencing an active orf outbreak when the sera were collected; in these cases, samples of scabs from orf-infected lesions were also collected in sterile tubes and stored at −80°C for subsequent virus isolation and PCR analysis. A total of five orf isolates were obtained from these scabs (data not shown). Three farms had never experienced an ORFV outbreak. Eight farms had experienced at least one ORFV outbreak within two years of sera collection, and an ORFV outbreak occurred at one farm just one month after sera collection. All sera were stored in PBS with 50% glycerol at −80°C until needed for analysis.

**Table 1:** Summary of Orf Titers in All Samples by Location.

Date of Collection	Sample Numbers	Location of Herd	Herd Size	# of Samples	# Positive	Positive (%)	Titer<2000	Titer = 2000	Titer = 4000	Titer = 8000	Titer = 16000	Species	Herd Infection Status	Notes
2011-12-18	1-1 to 1-15	Fuzhou, Zhuqi	70	15	13	86.7	2	4	5	3	1	Crossbred goats	Recovering	
2011-12-27	2-1 to 2-21	Fuzhou, Fuqing	300	21	15	71.4	6	4	8	2	1	Fuqing goats	Recovering	
2011-12-28	3-1 to 3-30	Fuzhou, Nantong	150	30	28	93.3	2	9	14	4	1	Crossbred goats	Recovering	
2011-12-30	4-1 to 4-21	Nanping, Zengcuo	85	21	21	100.0	0	2	8	4	7	Crossbred goats	Infected	virus isolated
2011-12-30	5-1 to 5-17	Nanping, Yangcuo	51	17	13	76.5	4	7	3	3	0	Crossbred goats	Recovering	
2011-12-31	6-1 to 6-30	Fuzhou, Nanyu	145	30	3	10.0	27	3	0	0	0	Crossbred goats	Healthy	
2012-1-8	7-1 to 7-43	Sanming, Guangming	120	43	2	4.7	41	1	0	1	0	Crossbred goats	Healthy	
2012-1-9	8-1 to 8-33	Sanming, Xiping	85	33	12	36.4	21	7	4	1	0	Crossbred goats	Healthy	
2012-2-21	9-1 to 9-5	Ningde,Zhongzhu	70	5	5	100.0	0	0	0	3	2	Crossbred goats	Infected	virus isolated
2012-3-8	10-1 to 10-14	Fuzhou, Dangshang	50	14	13	92.9	1	0	3	4	6	Crossbred goats	Infected	virus isolated
2012-3-1	11-1 to 11-32	Fuzhopu, Mingqing	78	32	23	71.9	9	13	5	5	0	Crossbred goats	Recovering	
2012-3-1	12-1 to 11-15	Fuzhou, Minqing	55	15	13	86.7	2	2	5	5	1	Crossbred goats	Recovering	
2012-3-1	13-1 to 13-52	Fuzhou, Mingqing	120	52	46	88.5	6	11	16	13	6	Crossbred goats	Recovering	
2012-4-22	14-1 t o 14-9	Fuzhou, Zhuxi	68	9	9	100.0	0	0	1	3	5	Crossbred goats	Infected	virus not isolated
2012-5-18	15-1 to 15-12	Fuzhou, Shangjie	120	12	9	75.0	3	2	1	5	1	Crossbred goats	Infected	virus isolated
	1-1 to 15-12	-	1567	349	225	64.5%	124	65	73	56	31			

### Enzyme-linked immunoassay (ELISA)

The test procedure and calculation of results of indirect ELISA were as described by Yirrell, Reid, Norval and Howie (1989), with some modifications. Briefly, the plates were coated with the purified native orf viral lysate (50 ng per well) by incubation at 4°C overnight. Plates were then washed before incubation with diluted samples. Test sera were diluted in a two-step process to a final, effective dilution of 1∶2000 to 1∶16000. The plates were then incubated for 1 h at 37°C, and then washed. The appropriate dilution of an HRP-conjugated rabbit anti-ovine Ig (Sigma) was then added. The plates were incubated 1 h at 37°C, and then washed. A 3′,5,5′-Tetramethylbenzidine (TMB) (Sigma) substrate was then added and reacted 10 minutes at 37°C. After using 2% H_2_SO_4_ to end the reaction, the plates were read at 450 nm in an iMark Microplate Reader (Bio-Rad, U.S.A.). On each plate, two duplicate wells of diluted positive serum (1∶2000) was used as a positive control. Additionally, one well of diluted non-immune serum (1∶2000) and one well of ELISA sample diluent (without serum) was included in each plate as a sero-negative control and blank, respectively. In each experiment, the ELISA optical density (O.D.) reading of the reference positive serum was at least twice that of the negative sera at the same dilution. Any O.D. reading of a diluted serum sample measured as at least twice that of the sero-negative control at the same dilution was regarded as a sero-positive detection.

### Western blot analysis

A total of 20 µg of purified native orf viral lysate was resolved by SDS-PAGE gel (10% acrylamide) and transferred by electroblotting to a nitrocellulose membrane, which was subsequently cut into multiple strips of approximately equal width. Strips were probed with goat sera (test sera). The strips were then incubated with HRP-conjugated rabbit anti-ovine IgG antibodies (Sigma) and washed before incubation with an ECL chemiluminescent substrate as a reporter (Pierce-Thermo Scientific, U.S.A.).

### DNA extraction from scab suspensions or cell-cultured virions

From December 31, 2011 to October 1, 2012, ten scabs from the outbreaks in ten different farms were collected, and eight virus strains have been isolated from them. The viral genomic DNAs were isolated from tissue suspensions or cell-cultured virions (200 µl each) by using QIAamp DNA blood kit (QIAGEN, Germany) following the manufacture's instructions. All animal protocols used in this study have been approved by the Department of Science and Technology of Fujian Province, and were in compliance with the guidelines of the Animal Care Committee, Fujian Agriculture and Forestry University (Certification Number: CNFJAC0027).

### Polymerase chain reaction (PCR) and DNA sequencing

**Figure 2:**
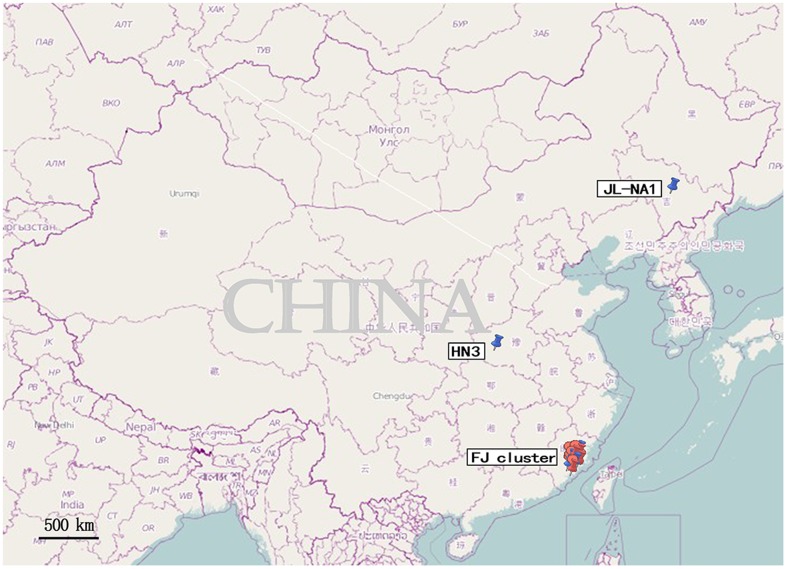
Sites in China at which orf isolate sequences of ORFV011 and ORFV059 genes were used for phylogenetic analysis. The following link is for Fig.2: http://www.openstreetmap.org/

Sites in China at which orf isolate sequences of ORFV011 and ORFV059 genes used for phylogenetic analysis are depicted in Figures 1 and 2 [16].

PCR was performed on DNA extracted from skin scabs or infected cell cultures. Two sets of primers were designed based on the genomic sequences of OV-IA82 to amplify the entire open reading frame of two highly conserved genes, ORFV011 (B2L) and ORFV059 (F1L). The primer sequences were as follows: ORFV011Fw1: 5′- AAGCGGAGACCTAATGCC-3′; ORFV011Rv1: 5′-ACCTTTCCCCAGAACCC-3′; ORFV059Fw1: 5′-CTCGGTCAAGGACTGGATA-3′; ORFV059Rv1: 5′-GATGGCTGGATGGTGCA-3′. PCR was carried out in a 50 µl reaction volume containing 10 µl of 5X PCR buffer (10 mM Tris-HCL and 50 mM KCL), 2 µl of DNA template, 200 µM dATP, dTTP, dCTP, dGTP, 1 µM of each primer, 25 µM MgCl2 and 0.5 µl of Taq polymerase (Takara Co., Japan). PCR was performed in a thermocycler (Bio-Rad, U.S.A.) for 35 cycles, consisting of denaturation at 98°C for 1 min, annealing at 58°C for 45 s and extension at 72°C for 90 s, and a final elongation of 10 min at 72°C. The amplified DNA products were resolved by 1% agarose gel electrophoresis and analyzed with an IS-1000 Digital Imaging System (Bio-Rad, U.S.A.). The amplified gene was purified using an agarose gel DNA extraction kit (OMEGA, U.S.A.) according to the manufacturer's instructions. Automated nucleotide sequencing of ORFV011 and ORFV059 was performed using 3730XL DNA Analyzer with the BigDye terminator v3.1 by Shanghai ShengGong Biological Engineering Technology Service Co., LTD, China.

## Results

### Indirect ELISA

Sera from a total of 349 goats from 15 farms were tested for antibodies reactive to native orf viral lysate proteins in this survey (Table 1). Sero-positive reactivity to the native orf lysate would be consistent with prior or active infection with ORFV. Based on statements of the farmers and our own investigations, the 15 farms were divided into three categories. In the first category, three herds that reportedly had never been infected by ORFV were classified as “healthy” herds. In the second category, the eight farms that suffered at least one ORFV outbreak within two years of sera collection were classified as recovering herds. In the third category, the four farms experiencing an orf disease outbreak contemporaneously with sera collection were classified as infected herds.

**Figure 3:**
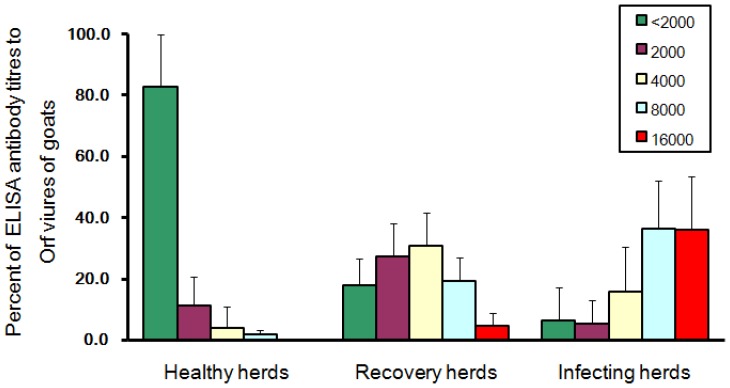
ELISA antibody titers to orf virus of goats from farms with different infection statuses in Fujian Province.

Table 1 illustrates the results of reactivity of test sera from goats at these 15 farms to purified orf viral lysates, as tested by indirect ELISA at dilution ratios of 1∶2000, 1∶4000, 1∶8000 and 1∶16000. From the three “healthy” herds, among the 106 goat sera tested, 16 percent were sero-positive for ORFV at one or more dilution ratios tested (see Table 2). In the recovering herds, among 182 goat sera tested, 83% were sero-positive for ORFV at one or more dilution ratios tested (see Table 3). In the infected herds, among 61 goat sera tested, 93.4% were sero-positive for ORFV at one or more dilution ratios tested (see Table 4). Figure 3 depicts differences in serum titers to orf virus in goats from the three categories of herd infection status. In the “healthy” herds, more than 80% of serum titers were less than 2000 (i.e., sero-negative at a dilution ratio of 1∶2000), and no test serum was sero-positive at a dilution ratio of 1∶16000. In the recovering herds, about 20% of serum titers were below 2000, about 30% of serum titers equaled 2000 (i.e., were sero-positive for ORFV only at the 1∶2000 dilution ratio), more than 30% of serum titers equaled 4000 (i.e., were sero-positive for ORFV at dilution ratios of 1∶4000 and 1∶2000), about 20% of serum titers were 8000 (i.e., were sero-positive for ORFV at dilutions ratios of ≤1∶8000), and only a few sera had titers of 16000 (i.e., were sero-positive for ORFV at all dilutions, including 1∶16000). By contrast, in the infected herds, nearly 80% of sera had titers of ≥8000, about 15% had titers of 4000, and less than 10% of serum titers were ≤2000.

**Table 2:** Summary of “Healthy” Herds.

Date of collection	Sample Numbers	Location of Herd	Herd Size	# of Samples	# Positive	Positive (%)	Titer<2000	Titer = 2000	Titer = 4000	Titer = 8000	Titer = 16000	Species	Herd Infection Status	Notes
2011-12-31	6-1 to 6-30	Fuzhou, Nanyu	145	30	3	10.0	27	3	0	0	0	Crossbred goats	Healthy	
2012-1-8	7-1 to 7-43	Sanming, Guangming	120	43	2	4.7	41	1	0	1	0	Crossbred goats	Healthy	
2012-1-9	8-1 to 8-33	Sanming, Xiping	85	33	12	36.4	21	7	4	1	0	Crossbred goats	Healthy	
			350	106	17	16.0	89	11	4	2	0		Healthy	
	6-1 to 8-33	-					84.0%	10.4%	3.8%	1.9%	0.0%		Healthy	

**Table 3:** Summary of Orf Titer in Recovering Herds.

Date of collection	Sample Numbers	Location of Herd	Herd Size	# of Samples	# Positive	Positive (%)	Titer<2000	Titer = 2000	Titer = 4000	Titer = 8000	Titer = 16000	Species	Herd Infection Status	Notes
2011-12-18	1-1 to 1-15	Fuzhou, Zhuqi	70	15	13	86.7	2	4	5	3	1	Crossbred goat	Recovering	
2011-12-27	2-1 to 2-21	Fuzhou, Fuqing	300	21	15	71.4	6	4	8	2	1	Fuqing goat	Recovering	
2011-12-28	3-1 to 3-30	Fuzhou, Nantong	150	30	28	93.3	2	9	14	4	1	Crossbred goat	Recovering	
2011-12-30	5-1 to 5-17	Nanping, Yangcuo	51	17	13	76.5	4	7	3	3	0	Crossbred goat	Recovering	
2012-3-1	11-1 to 11-32	Fuzhou, Mingqing	78	32	23	71.9	9	13	5	5	0	Crossbred goat	Recovering	
2012-3-1	12-1 to 11-15	Fuzhou, Mingqing	55	15	13	86.7	2	2	5	5	1	Crossbred goat	Recovering	
2012-3-1	13-1 to 13-52	Fuzhou, Mingqing	120	52	46	88.5	6	11	16	13	6	Crossbred goat	Recovering	
			253	182	151	83.0	31	50	56	35	10	Crossbred goat	Recovering	
							17.0%	27.5%	30.8%	19.2%	5.5%		Recovering	

**Table 4:** Summary of Orf Titers in Infected Herds.

Date of collection	Sample Numbers	Location of Herd	Herd Size	# of Samples	# Positive	Positive (%)	Titer<2000	Titer = 2000	Titer = 4000	Titer = 8000	Titer = 16000	Species	Herd Infection Status	Note
2011-12-30	4-1 to 4-21	Nanping, Zengcuo	85	21	21	100.0	0	2	8	4	7	Crossbred goat	Infected	virus isolated
2012-2-21	9-1 to 9-5	Ningde,Zhongzhu	70	5	5	100.0	0	0	0	3	2	Crossbred goat	Infected	virus isolated
2012-3-8	10-1 to 10-14	Fuzhou, Dangshnag	50	14	13	92.9	1	0	3	4	6	Crossbred goat	Infected	virus isolated
2012-4-22	14-1 to 14-9	Fuzhou, Zhuxi	68	9	9	100.0	0	0	1	3	5	Crossbred goat	Infected	virus not isolated
2012-5-18	15-1 to 15-12	Fuzhou, Shangjie	120	12	9	75.0	3	2	1	5	1	Crossbred goat	Infected	virus isolated
			393	61	57	93.4	4	4	13	19	21		Infected	
							6.6%	6.6%	21.3%	31.1%	34.4%		Infected	

**Figure 4:**
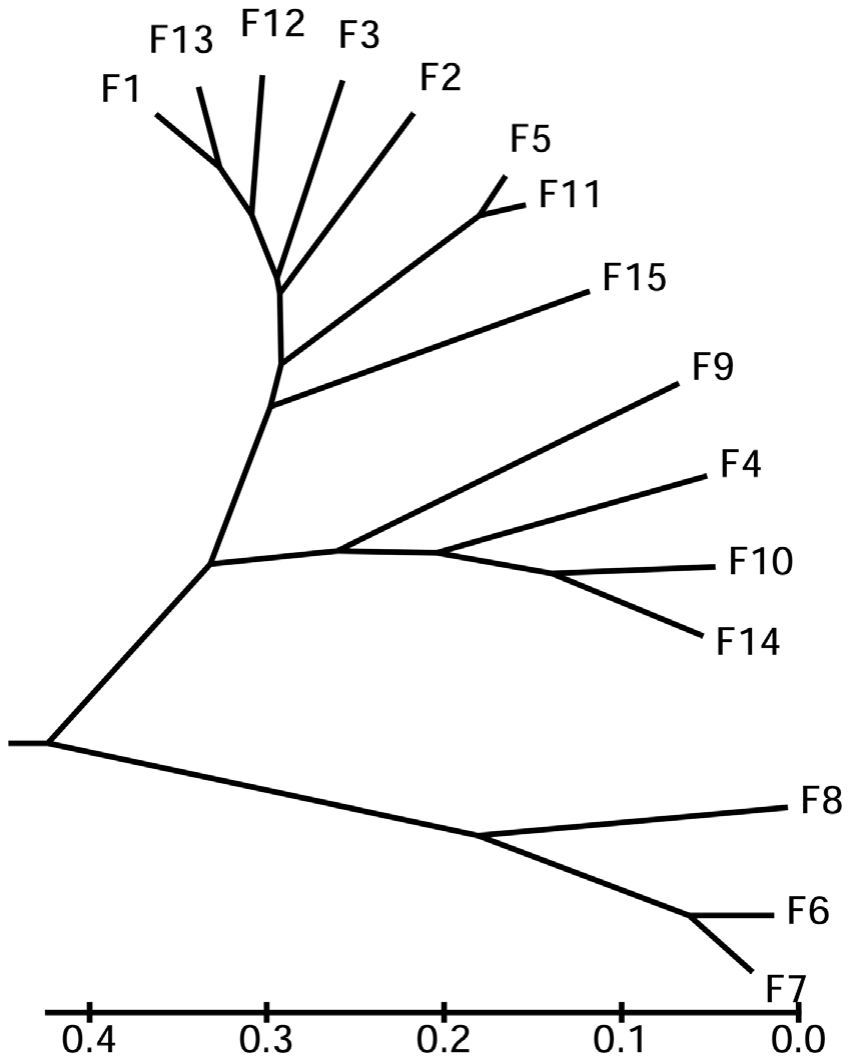
A serum titer clustering tree of 15 different farms based on average serum titers to orf. The phylogenetic relationship was constructed by using the NJ program of MEGA version 5.0.

To analyze the relationships of these serum titers, we calculated the distance between serum titers measured at each of 15 farms (Table 5) to construct a serum titer clustering tree (Figure 4). Interestingly, the serum titer clustering tree demonstrated that the 15 farm serum titers clustered into three branches: The three “healthy” herds all clustered together, all eight recovering herds share great homology and clustered together, and the four infected herds also clustered together. This further confirms specificity of the ELISA in our survey.

**Table 5:** Divergence of serum titers from 15 different farms with Distance Matrix.

	F1	F2	F3	F4	F5	F6	F7	F8	F9	F10	F11	F12	F13	F14	F15
F1	0.000														
F2	0.206	0.000													
F3	0.170	0.263	0.000												
F4	0.347	0.426	0.383	0.000											
F5	0.247	0.320	0.358	0.556	0.000										
F6	0.878	0.736	0.985	1.050	0.775	0.000									
F7	0.937	0.791	1.046	1.095	0.849	0.096	0.000								
F8	0.578	0.444	0.681	0.789	0.474	0.313	0.389	0.000							
F9	0.686	0.801	0.815	0.571	0.772	1.158	1.184	0.974	0.000						
F10	0.477	0.540	0.577	0.245	0.627	1.004	1.038	0.788	0.388	0.000					
F11	0.281	0.321	0.394	0.582	0.054	0.725	0.800	0.425	0.791	0.643	0.000				
F12	0.189	0.293	0.302	0.336	0.376	0.903	0.947	0.633	0.573	0.411	0.404	0.000			
F13	0.094	0.252	0.236	0.289	0.301	0.894	0.947	0.606	0.597	0.393	0.332	0.128	0.000		
F14	0.629	0.711	0.730	0.389	0.750	1.119	1.152	0.922	0.328	0.185	0.766	0.569	0.545	0.000	
F15	0.365	0.442	0.530	0.519	0.366	0.784	0.827	0.556	0.481	0.462	0.372	0.291	0.315	0.567	0.000

### Western blot analysis

**Figure 5:**
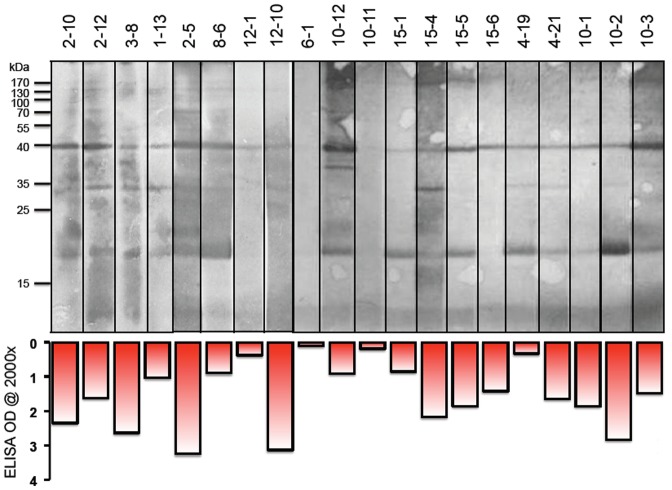
Western blot analysis. Orf virus antigens were probed with different sera from 9 farms with different antibodies titers.

Autoradiographs prepared using sera from nine farms are presented in Figure 5. Lane 9 contains serum from a “healthy” herd, and lane 11 represents serum from an infected herd. Both of them had serum titers of <2000 as determined by indirect ELISA and reacted with none of the purified native orf viral protein bands in the Western blot. However, a band with an approximate molecular weight (MW) of 40 kDa is evident in the other lanes, all of which correspond to sera that also reacted positively to the orf viral lysate by indirect ELISA. Most of positive sera in this study also reacted with a band of an approximate MW of 18 kDa. In some samples, 35 kDa, 38 kDa and 130 kDa bands were also detectable. However, the autoradiographic images of these bands were less intense than the 40 kDa and 18 kDa bands. Sera from nine farm animals that demonstrated negative reactivity by indirect ELISA also generated no detectable bands in the Western blot. Moreover, all sera demonstrating positive detection via indirect ELISA also identified a 40 kDa protein in the Western blot, validating the reliability of ELISA test results.

### PCR amplification

PCR amplified full length transcripts of the ORFV011 and ORFV059 genes in eight cell culture isolates (ORFV-FJ-GO, ORFV-FJ-GT, ORFV-FJ-DS, ORFV-FJ-SL, ORFV-FJ-SJ1, ORFV-FJ-YX, ORFV-FJ-NP, ORFV-FJ-FQ) and three scabs samples (ORFV-FJ-GS, ORFV-FJ-ZX, ORFV-FJ-SJ2). The sizes of products were approximately 1460 bp (ORFV011, an open reading frame of 1,137 bp) and 1268 bp (ORFV059, an open reading frame of 1023 bp) (data not shown). The identity of each PCR product was confirmed by automated DNA sequencing. The sequences were edited, aligned, and submitted to GenBank with the following Accession Numbers:

ORFV011-FJ-DS:KC568390; ORFV011-FJ-FQ:KC568391; ORFV011-FJ-GO:KC568392; ORFV011-FJ-GS:KC568393; ORFV011-FJ-GT:KC568394; ORFV011-FJ-NP:KC568395; ORFV011-FJ-SJ1:KC568396; ORFV011-FJ-SJ2:KC568397; ORFV011-FJ-SL:KC568398; ORFV011-FJ-YX:KC568399; ORFV011-FJ-ZX:KC568400; ORFV059-FJ-DS:KC568401; ORFV059-FJ-FQ:KC568402; ORFV059-FJ-GO:KC568403; ORFV059-FJ-GS:KC568404; ORFV059-FJ-GT:KC568405; ORFV059-FJ-NP:KC568406; ORFV059-FJ-SJ1:KC568407; ORFV059-FJ-SJ2:KC568408; ORFV059-FJ-SL:KC568409; ORFV059-FJ-YX:KC568410; ORFV059-FJ-ZX:KC568411.

### Phylogenetic analysis

DNA sequences comparisons of the full length ORFV011 and ORFV059 sequences obtained by PCR amplification were conducted to determine the phylogenetic relationships between isolates. Using GenBank nucleotide sequences of either ORFV011, ORFV059 or both, the phylogenetic relationships between 11 various Fujian isolates and with other ORFV isolates was constructed. The phylogenetic analysis was performed using Molecular Evolutionary Genetics Analysis (MEGA) 5.0 software, with bootstrap values calculated from 1000 replicates. The phylogenetic algorithm used for the building of the tree was the Maximum Likelihood Methods.

**Figure 6:**
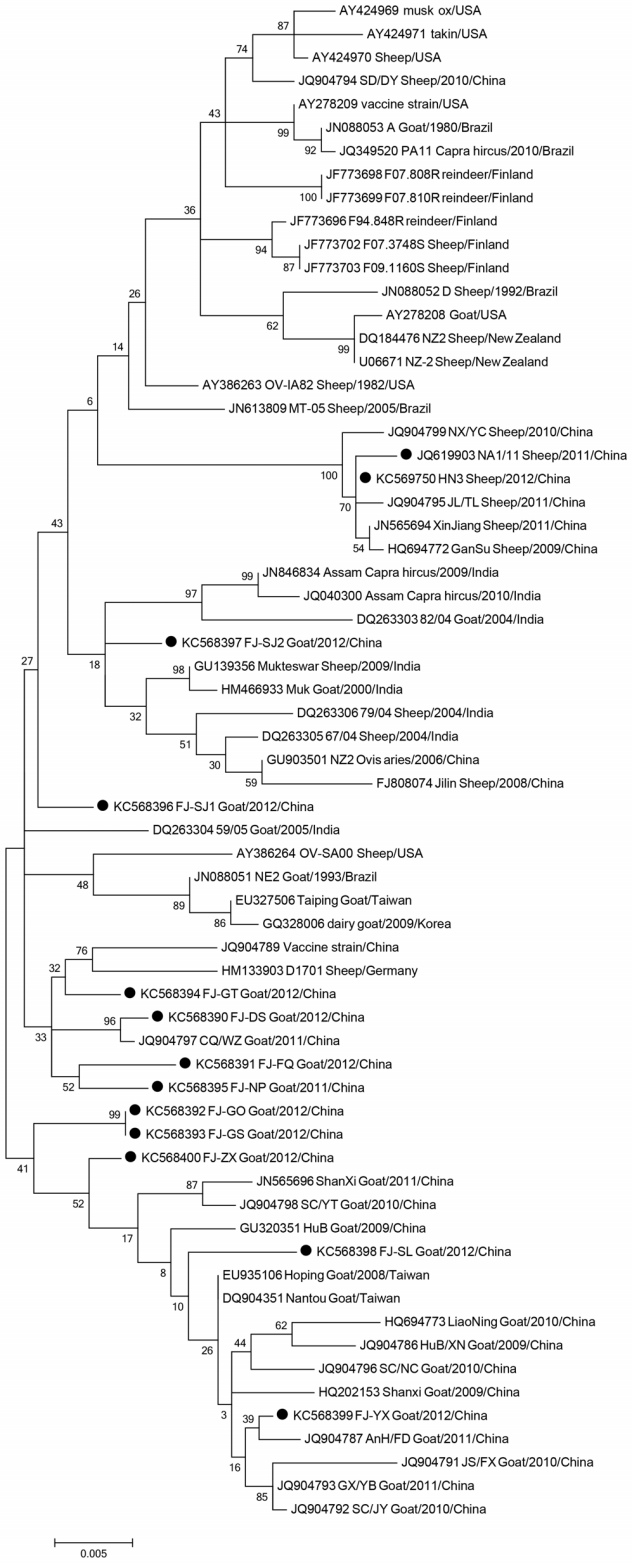
Phylogenetic analysis based on nucleotide sequences of ORFV011. The phylogenetic relationship was constructed by the Maximum Likelihood Method using MEGA 5.0 software. Black circles: 11 different orf isolates from Fujian Province (China) and two orf isolates from other provinces.

The phylogenetic analysis of 11 Fujian Province (China) ORFV 011 sequences (Figure 6) shows that they do not cluster together. Among the 11 Fujian ORFV sequences, only FJ-GO (KC568392) and FJ-GS (KC568393) shared 100% nucleotide identities, suggesting that they are the same virus, and the other nine sequences shared 98.2%–99.3% nucleotide identity. Interestingly, FJ-SJ1 (KC568407) and FJ-SJ2 (568408) shared 99% identity at the nucleotide level, despite the fact that they came from scabs collected from the same farm eight months apart. This suggests that the evolution of ORFV is rapid.

**Figure 7:**
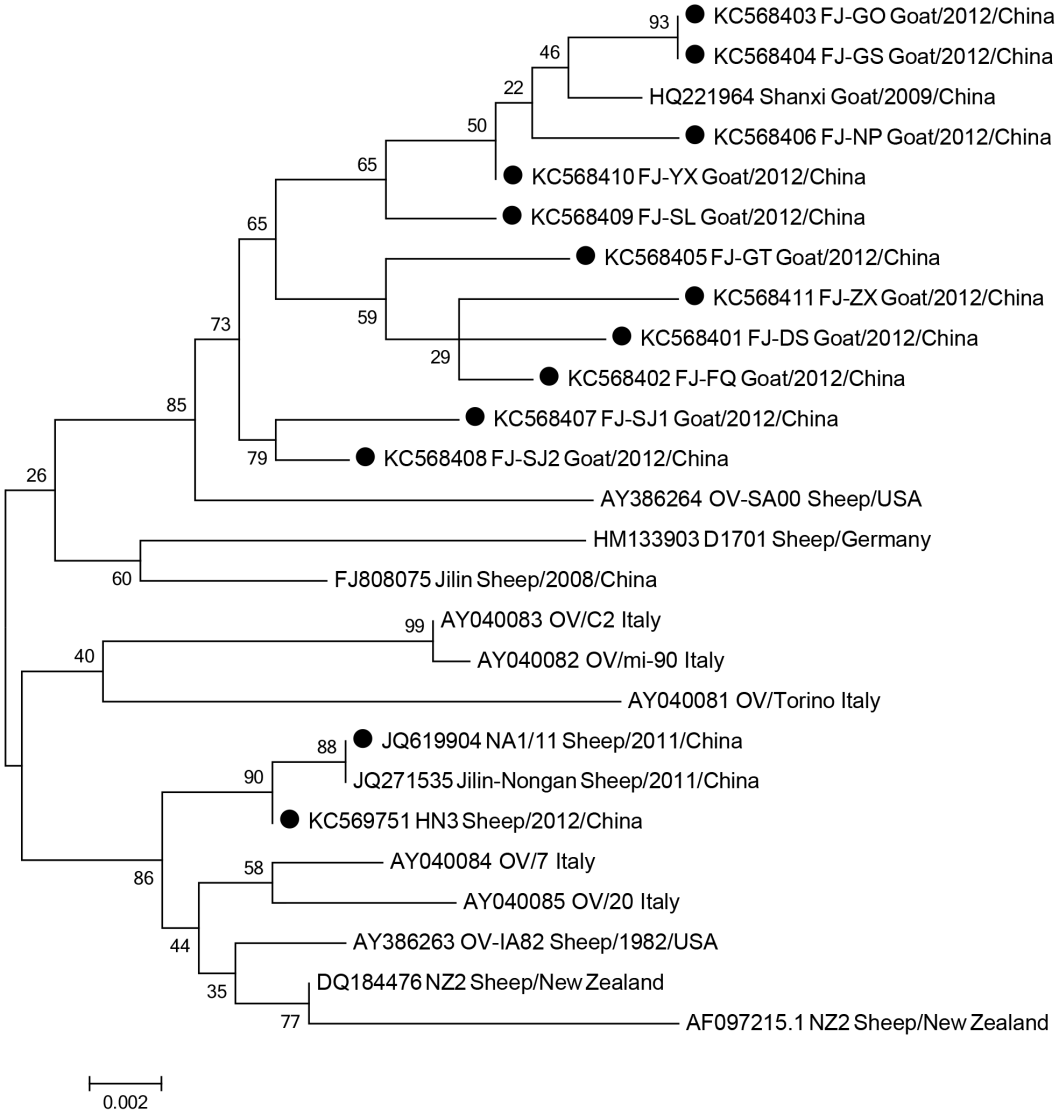
Phylogenetic analysis based on nucleotide sequences of ORFV059. The phylogenetic relationship was constructed by Maximum Likelihood Method using MEGA 5.0 software. Black circles: 11 different orf isolates from Fujian Province (China) and two orf isolates from other provinces.

The phylogenetic analysis of 11 Fujian Province (China) ORFV 059 sequences (Figure 7) shows that they nearly cluster together. Among 11 Fujian ORFV sequences, FJ-GO (KC568403) and FJ-GS (KC568404) shared 100% nucleotide identities, further suggesting they are the same virus, and the other 9 sequences shared 95.9%–99.3% nucleotide identity. FJ-SJ1 (KC568396) and FJ-SJ2 (KC568397) shared 98.9% similarities at the nucleotide level.

**Figure 8:**
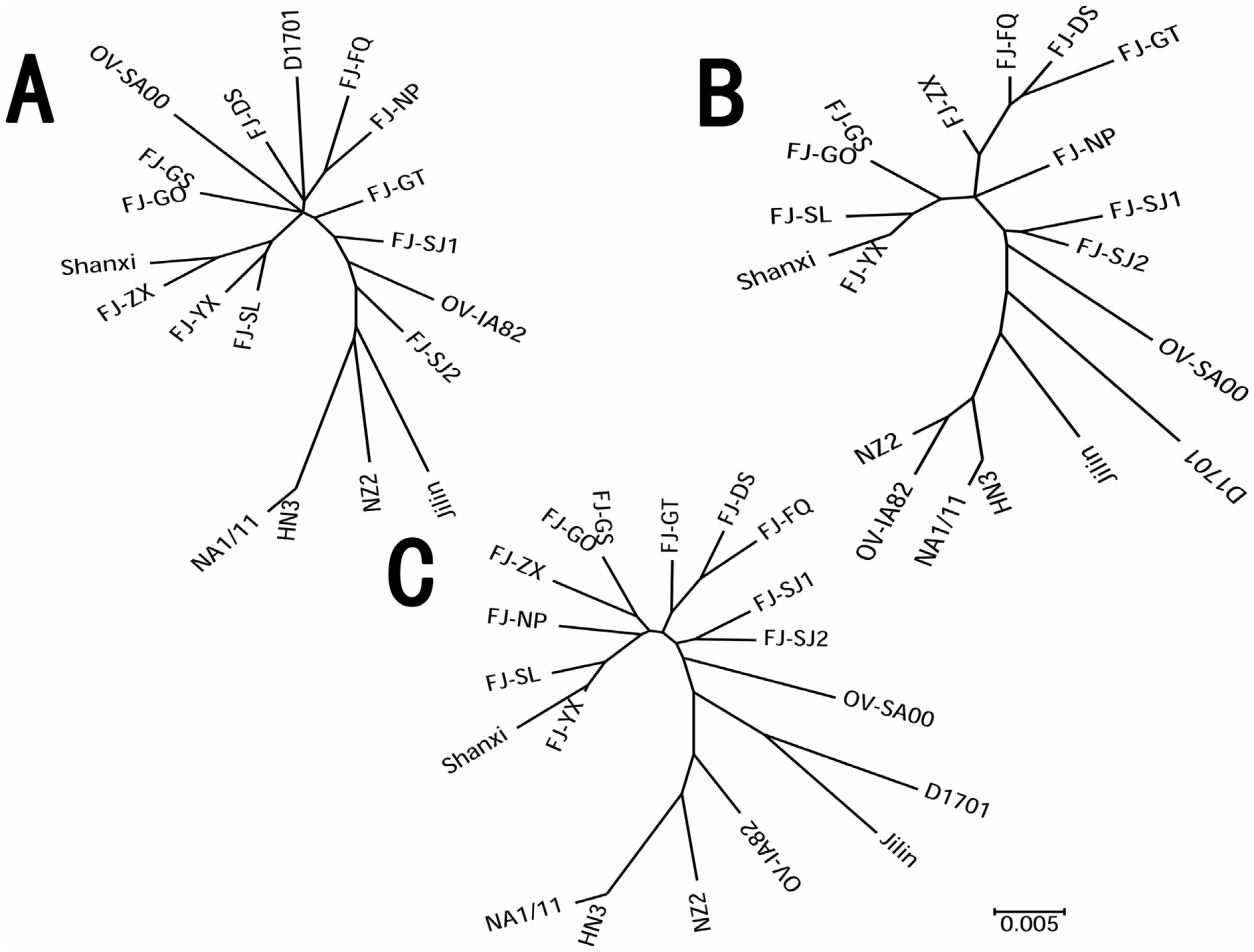
Phylogenetic analysis based on nucleotide sequences of ORFV011. (A). Phylogenetic analysis based on nucleotide sequences of ORFV059 (B).The phylogenetic based on nucleotide sequences of ORFV059(C) based on ORFV011 and ORFV059 with concatenated distance using MEGA 5.0. Tree was generated using by Maximum Likelihood Method using MEGA 5.0 software.

Phylogenetic analysis based on nucleotide sequences of ORFV011 (Figure 8A) shows Fujian Province 11 isolates do not cluster together. However, based on nucleotide sequences of ORFV0059 (Figure 8 B), the 11 Fujian Province isolates cluster together. If the phylogenetic analysis is based on the concatenated distances between the nucleotide sequences of ORFV011-059,(Figure 8C), the 11 Fujian Province isolates also cluster together. This reveals phylogenetic based on single gene maybe have some bias.

**Figure 9:**
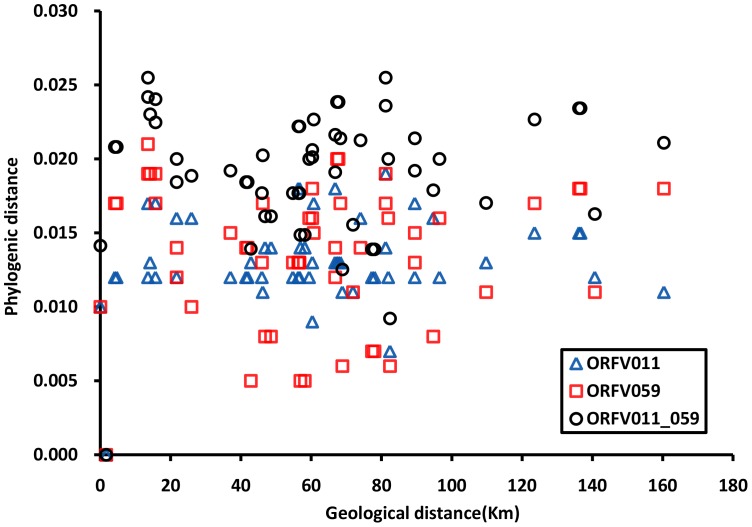
Ground-distance comparison based on ORFV011, ORFV059 and ORFV011-059 (with Euclidian distance). The y axis is the phylogenetic distance calculated by MEGA5, defined as substitutions per base. The x-axis is the distance in km between any pair of sample locations (as calculated by Google Maps). Three symbols represent phylogenetic distances based on ORFV011, ORFV059 and both sequences (ORFV011-059).

To compare and determine the phylogenetic relationship of 11 Fujian isolates, we drew a ground-distance comparison based on ORFV011?ORFV059 and ORFV011-059, where the Euclidian distance (D011-059)  =  ((D011)?2+(D059)?2)?0.5) (Figure 9). The data indicate that there is no correlation between geographical distance and phylogenetic distance.

## Discussion

Orf is an epitheliotropic disease with a worldwide distribution. In recent years, ORFV outbreaks occurred in many countries, including Greece (2003, 2004), Korea (2009), Brazil (2005, 2009), India (2006, 2009, 2010), Taiwan (2007), and Japan. In China between 2000 and 2010, ORFV outbreaks occurred in the following Provinces: Inner Mongolia (June 2005), Guangxi (March 2005), Shanxi (April 2005), Fujian (August 2005), Jilin (March 2006), Hubei (March 2009), and Jilin (November 2011). However, there is a lack of current epidemiological surveys of ORFV; 1992 is the last year for which data of such survey data is available [17]. Given the persistence and recurring nature of the disease and the fact that the vaccine is not widely used, it is suspected that ORFV outbreaks often go unreported and that the actual prevalence of ORFV infections among livestock herds is greatly under estimated. In this study, from a total of 349 goats from 15 different farms in Fujian Province, 225 (64.47%) were sero-positive for ORFV with a serum titer of ≥2000 as the positive threshold. Among the 15 different farms in our survey, only three farms did not report an infection with ORFV in the previous two years, eight farms reported at least one recent ORFV outbreak, and four farms were experiencing an outbreak of the disease at the time of sera collection. This was consistent with both our Western blot and indirect ELISA results; thus, the incidence of sero-positive farms in our survey is 80% (12/15). We found ten farms that had experienced ORFV outbreaks in Fujian Province of China between December 2011 and October 2012. This suggests that orf virus infections are ubiquitous in goat farms in this district. Orf virus can infect humans, with farmers, abattoir workers, veterinarians and shearers considered to be at greatest risk [18], [19]. In these ten infected farms (defined as those farms experiencing two or more distinct outbreaks during the survey period), we interviewed farmers who had direct with affected animals, however, there were no reports of human infection. In these cases, we also noticed these farmers were taking the precaution of washing their hands with alexipharmic water after handling affected animals. It is suspected that this is the reason why there were no human infections.ORFV011 (a homologue of vaccinia virus major envelope antigen p37K) encodes the envelope immunogenic protein [20], while the ORFV059 gene encodes an immunogenic protein and plays a role in virus mature and adsorption [21]. In the ORFV genome, both of them are conserved and have been widely used as a molecular targets for orf detection, epidemiological and phylogenetic analyses of PPVs [3].

In this study, we used complete ORFV011 and ORFV059 gene sequences from a total of 11 orf isolates from Fujian Province, China, and other sources world-wide for phylogenetic analysis. We found that, based on ORFV059, the 11 different ORFV isolates clustered together and that only isolates from the two flocks located 1 kilometer away from each other showed 100% identity at the nucleotide level, suggesting similar origin. OV-YX shares higher similarity with HQ221984.1 China Shanxi. According to the phylogenetic analyses based on ORFV011, the 11 orf isolate sequences do not cluster.OV-YX and OV-ZX also share higher similarity with the other orf isolates from worldwide, and OV-FQ and OV-NP are close to each other. Notably, OV-GO and OV-GS are identical at the nucleotide level, suggesting these two isolates are from the same origin. The other eight isolates are highly divergent, indicating considerable heterogeneity among orf virus isolates from goats in Northern Fujian, China.

The Western blot results showed that there are several orf virus antigens that appear to be immunogenic. One strong band about 40 kDa was recognized in all animals that were found to be sero-positive by indirect ELISA, but this band was absent when probed with sera from healthy animals. McKeever et al (1987) found all the sera used to probe Western blots also recognized the 40 kDa component of their test virus [22]. This implies that we may use this band in the future to judge if tested animals have been infected with orf virus.

## Conclusions

In summary, orf virus infections are ubiquitous in Fujian Province goat flocks without much attention being given to this fact by veterinary health care officials. Vaccination has not been implemented in this region to control the disease, despite the fact that significant veterinary and economic losses are caused by ORFV outbreaks. Phylogenetic analysis based on ORFV011 and ORF059 genes revealed that ten isolates are highly divergent, suggesting there is considerable heterogeneity among orf virus isolates from Fujian Province in China. Therefore we need further complete genome comparisons to further understand the diversity and biology of orf virus isolates in this region.
